# Identifying sarcopenia in advanced non‐small cell lung cancer patients using skeletal muscle CT radiomics and machine learning

**DOI:** 10.1111/1759-7714.13598

**Published:** 2020-08-06

**Authors:** Xing Dong, Xu Dan, Ao Yawen, Xu Haibo, Li Huan, Tu Mengqi, Chen Linglong, Ruan Zhao

**Affiliations:** ^1^ Department of Radiology Zhongnan Hospital of Wuhan University Wuhan China

**Keywords:** Computed tomography, lightGBM, machine learning, non‐small cell lung cancer, radiomics

## Abstract

**Background:**

Sarcopenia has been confirmed as a poor prognostic indicator of lung cancer. However, the lack of abdominal computed tomography (CT) hindered the application to assess the status of sarcopenia. The purpose of this study was to assess the ability of chest CT radiomics combined with machine learning classifiers to identify sarcopenia in advanced non‐small cell lung cancer (NSCLC) patients.

**Methods:**

This study retrospectively analyzed CT images of 99 patients with NSCLC. Skeletal muscle radiomics were extracted from a single axial slice of the chest CT scan at the 12th thoracic vertebrae level. In total, 854 radiomic and clinical features were obtained from each patient. Feature selection was conducted with FeatureSelector module, optimal key features were fed into the lightGBM classifier for model construction, and Bayesian optimization was adopted to tune hyperparameters. The model's performance was evaluated by specificity, sensitivity, accuracy, precision, F1‐score, Matthew's correlation coefficient (MCC), Cohen's kappa coefficient (Kappa), and AUC.

**Results:**

A total of 40 patients were found to have sarcopenia. Five optimal features were selected. In the base lightGBM model, the specificity, sensitivity, accuracy, precision, F1‐score, AUC, MCC, Kappa of validation set were 0.889, 0.750, 0.833, 0.818, 0.783, 0.819, 0.649, 0.648, respectively. After Bayesian hyperparameter tuning, the optimized lightGBM model achieved better prediction performance, and the corresponding values were 0.944, 0.833, 0.900, 0.909, 0.870, 0.889, 0.791, 0.789, respectively.

**Conclusions:**

Chest CT‐based radiomics has the potential to identify sarcopenia in NSCLC patients with the lightGBM classifier, and the optimal lightGBM model via Bayesian hyperparameter tuning demonstrated better performance.

**Key points:**

**Significant findings of the study:**

Our study demonstrates that chest CT‐based radiomics combined with lightGBM classifier has the ability to identify sarcopenia in NSCLC patients.

**What this study adds:**

Skeletal muscle radiomics would be a potential biomarker for sarcopenia identity in NSCLC patients.

## Graphical abstract

Our study demonstrates that chest CT‐based radiomics combined with lightGBM classifier has the ability in identifying sarcopenia in NSCLC patients, and skeletal muscle radiomics would be a potential biomarker for sarcopenia identity in NSCLC patients.

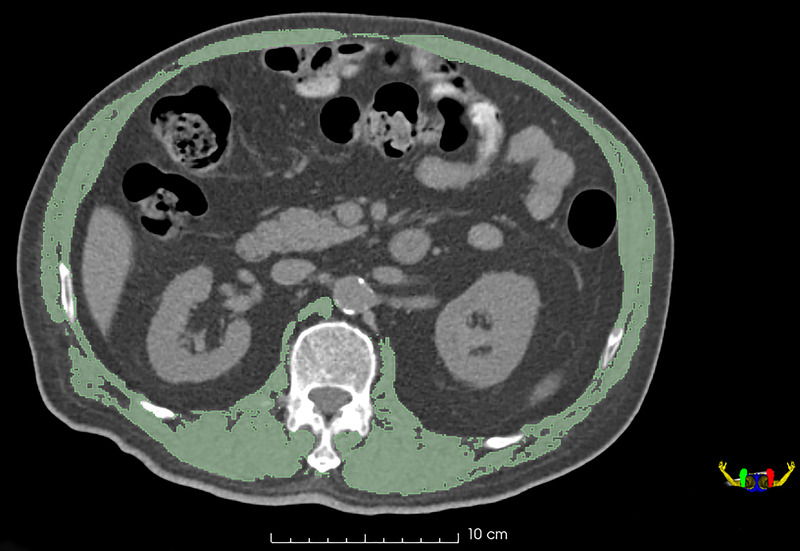

## Introduction

Globally, lung cancer is a significant factor for the fatalities caused by cancer, and non‐small cell lung cancer (NSCLC) constitutes roughly 75% to 80% of primary lung cancer.^1^ The TNM staging system can be applied to determine the postoperative prognosis for NSCLC patients. Nevertheless, the determinants of prognosis include more than tumor‐specific factors, and physical factors, such as body mass index and performance status, are also influential.^2^

Defined as the physical component of syndromes characterized by the significant loss of skeletal muscle mass and function, sarcopenia can result in physical disability, declining quality of life, and death.^3^ At present, it has been confirmed as a poor prognostic indicator of various cancers.^1^ In lung cancer, sarcopenia is associated with shorter survival, decreased tolerance to chemotherapy, and diminished functional ability. Thus, identifying NSCLC patients under threat of sarcopenia is very important.

Although various techniques such as D3‐Creatine dilution, dual‐energy x‐ray absorptiometry (DXA), computed tomography (CT), magnetic resonance imaging (MRI), and bioimpedance analysis (BIA) can be applied to identify sarcopenia by estimating the skeletal muscle mass (SMM), the CT or MR‐based identification and quantification of the skeletal muscle area is recommended, due to its precise differentiation between muscle, fat and other tissues and easily accessed in the clinical activity. Also, the single cross‐sectional area of muscle on abdominal CT or MRI at the third lumbar vertebra (L3) are considered gold standards for estimating body composition, as it is linearly correlated to whole body skeletal muscle mass, and currently used to define sarcopenia in trials.^1^, ^2^, ^4^

Chest CT is considered as the standard method employed to diagnose and follow‐up lung cancer; however, it has rarely extended to the L3 level.^2^, ^5^ In recent research with the majority of patients suffering lung cancer, only 65% received useful scans to assess SMM at the L3 level, hindering its application to assess the status of sarcopenia in those patients.^6^

Radiomics, defined as an algorithm‐based large scale quantitative analysis of imaging features, can reveal disease features and reflect underlying pathophysiology.^7^ Together with machine‐learning (ML) models which are data‐driven analysis methods for mining implicit clinical values of these image features, radiomics have yielded promising results, such as survival, tumor progression, genetic mutations or expression profiles.^8^ Recently, de Jong *et al*.^9^ applied skeletal muscle CT radiomics to predict the response to chemotherapy in stage IV NSCLC, showing the prospects of skeletal muscle CT radiomics in the diagnosis of muscle loss in NSCLC patients. However, no research as yet to date has identified sarcopenia in cancer patients based on radiomics and ML operations.

In this study, we extracted skeletal muscle radiomic features from chest CT scans at the 12th thoracic vertebrae (T12) level and employed a powerful gradient boosting decision machine (Light Gradient Boosting Machine [LightGBM]) to determine the value of chest skeletal muscle CT radiomics in different sarcopenia and nonsarcopenia advanced NSCLC patients.

## Methods

### Patient characteristics

The study was approved by the Ethics Committee of our institution, and the requirement for informed consent was relinquished. A total of 107 patients with suspected lung cancer who had undergone ^18^F‐FDG PET/CT at our hospital between July 2017 and March 2019 were recruited into this study. The inclusion criteria included: (i) whole‐body PET/CT images available before treatment; (ii) histologically diagnosed NSCLC and clinically confirmed primary advanced NSCLC (stage IIIB, IV); (iii) ≥18 years of age; (iv) complete clinical information such as height, weight, pathology, and TMT staging; and (v) without the comorbidity history of other malignant tumors.

The exclusion criteria included: (i) metastases in the chest or abdominal wall in the ROI (*N* = 2); (ii) poor quality chest and abdominal CT image (*N* = 1); (iii) only contrast‐enhanced CT imaging (*N* = 5). The TNM staging complies with the eighth edition of the IASLC (The International Association for the Study of Lung Cancer) NSCLC (non‐small cell lung cancer) TNM staging standards.

Finally, the total number of patients satisfying the criteria were randomly classified to the training and validation datasets at a ratio of 7:3, and their CT images were retrospectively analyzed.

### Image acquisition

Noncontrast‐enhanced CT data were obtained from pretherapeutic whole‐body (from the skull to femurs) PET/CT studies with the Biograph mCT PET/CT system (S64, Siemens Co., Erlangen, Germany). CT was performed with normal shallow respiration under a low‐dose setting (120 kV, 80 mAs), an axial field of view of 21.6 cm, as well as a three‐dimensional model. This experimental process was operated in the B30f convolution kernel with a slice thickness of 3.0 mm and a matrix size of 512 × 512.

### Body composition analysis

The skeletal muscle index (SMI, cm^2^/m^2^) was utilized to evaluate body composition. For SMI calculation, the total cross‐sectional area (CSA, cm^2^) of skeletal muscle was divided by the square of patient height (m^2^), at the third lumbar (L3) vertebrae level.

**Figure 1:**
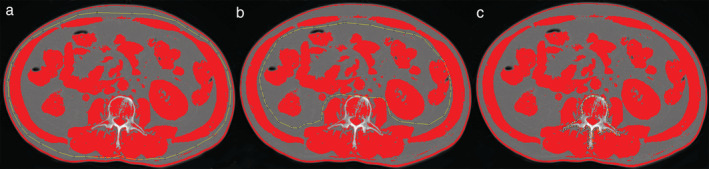
The skeletal muscle CSA measurement at the L3 level with NIH ImageJ software. (**a**) The outer area for skeletal muscle mass; (**b**) inner area for skeletal muscle mass; and (c) area within the vertebral body. The skeletal muscle CSA was calculated as the formula: (outer area − inner area − vertebral body area)/100. CSA, cross‐sectional area. L3, third lumbar.

To measurement the CSA, the center‐most CT slice within the L3 vertebral level was chosen and exported into the open‐source image processing program NIH ImageJ software (version 1.52n) for further semi‐automated image analysis, and the Hounsfield unit ranges constructed for skeletal muscle (−29 to 150) were selected. First, the outer area for SMM was obtained, then the inner area for SMM, and the area within the vertebral body, respectively, as shown in Fig 1. The skeletal muscle CSA was calculated as the formula: (outer area−inner area−vertebral body area)/100. The measurements are detailed in the literature.^10^, ^11^

Sarcopenia is defined in men as L3 lumbar SMI <43 cm^2^/m^2^ if BMI < 25 kg/m^2^ and SMI index <53 cm^2^/m^2^ if BMI ≥ 25 kg/m^2^, and in women as SMI < 41 cm^2^/m^2^.^12^, ^13^

### Region‐of‐interest segmentation and radiomic feature extraction

**Figure 2:**
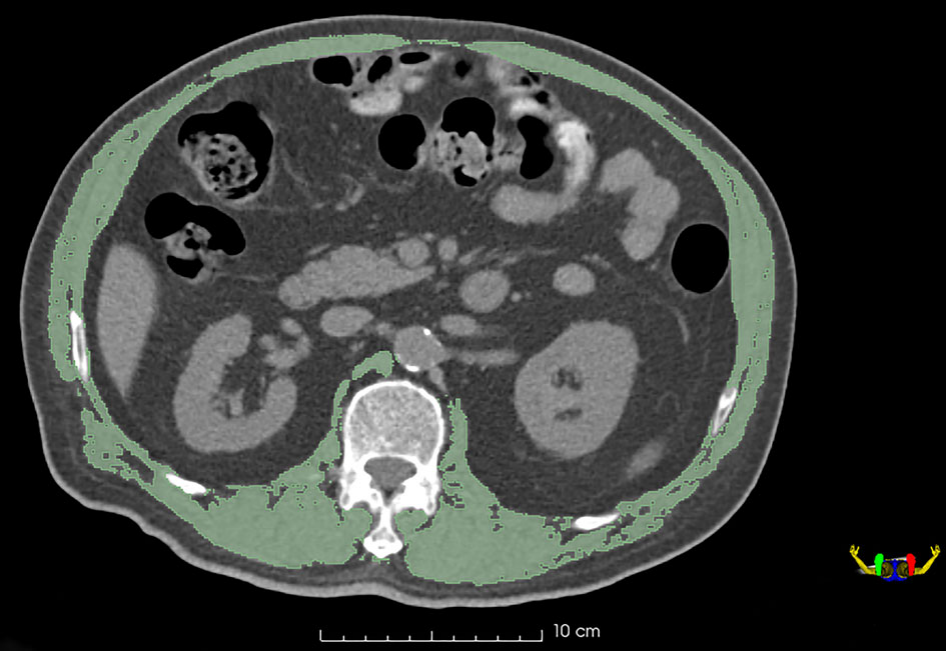
Skeletal muscle semi‐automated segmentation utilizing 3D‐Slicer Software at 12th thoracic vertebra level.

We performed semi‐automated segmentation utilizing 3D‐Slicer Software (version 4.11.0, www.slicer.org). The region of interest (ROI) of skeletal muscles was performed on a single axial slice of the CT scan at the center of the 12th thoracic (T12) vertebra level. Skeletal muscle was delineated with the same thresholds of Hounsfield units as previously described in body composition analysis, and falsely demarcated structures such as the vertebrae, ribs, fat, and organs, were manually corrected, as shown in Fig 2.

Radiomic feature extraction was carried out using the “radiomics” extension within 3D‐Slicer which is a convenient front‐end interface of PyRadiomics (version 2.2.0). As a flexible open‐source platform, PyRadiomics is not only effective in the extraction of a large‐size panel of engineered features from medical images, but is also capable of making feature definitions and image processing standardized.^14^, ^15^

Before the extraction of radiomic features, the CT images were processed. Preprocessing consisted of the process to discretize the image with a fixed bin width of 25 Hounsfield units and resample it to a voxel size of 1 × 1 × 1 mm^3^. A built‐in filter (wavelet) was used for every single ROI, and based on which 851 radiomic features were obtained, including 14 shape features, 18 first‐order intensity statistics features, 75 texture features, and 744 wavelet features. Please refer to the Supporting Information Table S1 for the specific list of extracted features. The definition of the mentioned radiomic features is available at http://pyradiomics.readthedocs.io/en/latest/features.html.

**Figure 3:**
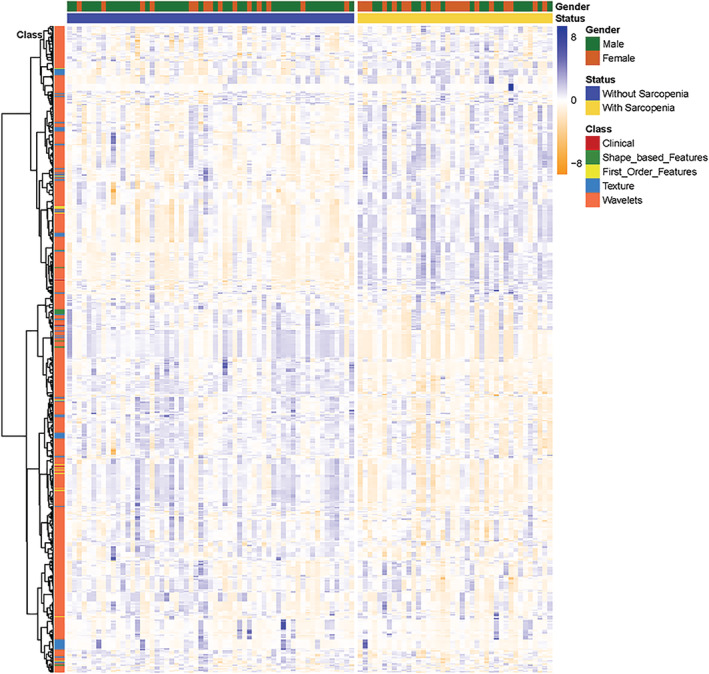
Heatmaps of the radiomic and clinical features for 99 patients.

In total, 854 features were obtained for every patient: 851 radiomic features from the ROI and three clinical features including the subject's age, gender, BMI, as illustrated in Fig. 3.

### Robustness analysis

Both the body composition analysis and the radiomic features were generated by one musculoskeletal radiologist who had eight years of experience, and who chose 30 random images. The same procedure was repeated one week later for intraobserver reproducibility assessment. To assess interobserver reproducibility, the 30 random images were evaluated by another radiologist independently.

Intra‐ and interclass correlation coefficients (ICCs) were adopted to assess inter‐ and intraobserver reproducibility of the body composition analysis and ROI delineation. ICCs over 0.75 were considered high agreement. After the stable radiomic features were selected, z‐score normalization ([value – mean value]/standard deviation) was performed for further analysis.

### Machine learning methods

#### Feature selection

In the machine learning pipeline, feature selection plays a vital role in identifying the most significant features in a dataset. Due to unnecessary features, training speed is reduced, model interpretability deteriorates, and, most crucially, the performance in generalization on the test set is compromised.

We used the FeatureSelector module (https://github.com/WillKoehrsen/feature-selector) for selecting features to remove from the training dataset. This module takes five different approaches to filter the features:

1. *Identify_missing values*. Features with a missing fraction greater than 0.60 were removed.
2. *Identify_ single_unique values*. The features exhibiting only a single unique value were removed.
3. *Identify_ collinear values*. Pairs of collinear features based on the Pearson correlation coefficient were found and one feature randomly excluded with a correlation value higher than a given threshold, which was set as 0.80 in this study.
4. *Identify_ zero_importance values*. This method identifies the features with zero importance to the given set of features with the Gradient Boosting Machine (GBM) learning model.
5. *Identify _low_importance values*. The features with no contribution were removed to define cumulative feature importance from the GBM. The features that are the least important and redundant for reaching 90% of the overall feature importance can then be identified.

Because the number of features from zero_importance and low_importance may vary due to training a model multiple times, we had re‐run several iterations for optimization.

#### Machine learning methods and hyperparameter tuning

After the redundant features were removed, the remaining features were fed into the LightGBM classifier to train the classification scheme to identify the sarcopenia subjects. LightGBM refers to a novel Gradient Boosting Decision Tree extension proposed by Microsoft.^16^

To improve the predictive performance of the base models and avoid potential overfitting, a Bayesian optimization library termed as Optuna was adopted in this study to efficiently tune hyperparameters and experimentally benchmark its performance. Optuna (https://github.com/pfnet/optuna) is a next‐generation framework designed for the automation and acceleration of the hyperparameters optimization studies,^17^ which searches the hyperparameter space for optimal combinations of the hyperparameters.

In all the optimizations, the number of Bayesian optimization trials was 100, Matthew's correlation coefficient (MCC) was chosen as the metric to be maximized, and 11 lightGBM hyperparameters were optimized (Table S2). A detailed description of these hyperparameters can be found elsewhere (https://lightgbm.readthedocs.io/en/latest).

### Performance evaluation

Seven measures were used to assess the performance of the method suggested in this paper. There were specificity (SP), sensitivity (SN), accuracy (ACC), precision (PRE), F1‐score, Matthew's correlation coefficient (MCC) and Cohen's kappa coefficient (Kappa), and are defined as follows:

(1) $$\mathrm{SP}=\frac{\mathrm{TN}}{\mathrm{TN}+\mathrm{FP}}$$

(2) $$\mathrm{SN}=\frac{\mathrm{TP}}{\mathrm{TP}+\mathrm{FN}}$$

(3) $$\mathrm{ACC}=\frac{\mathrm{TP}+\mathrm{TN}}{\mathrm{TP}+\mathrm{TN}+\text{FT}+\mathrm{FN}}$$

(4) $$\mathrm{PRE}=\frac{\mathrm{TP}}{\mathrm{TP}+\mathrm{FP}}$$

(5) $$F1\;\text{score}=2\times \frac{\mathrm{TP}}{2\mathrm{TP}+\mathrm{FP}+\mathrm{FN}}$$

(6) $$\mathrm{MCC}=\frac{\left(\mathrm{TP}\times \mathrm{TN}\right)-\left(\mathrm{FP}\times \mathrm{FN}\right)}{\sqrt{\left(\mathrm{TP}+\mathrm{FP}\right)\left(\mathrm{TP}+\mathrm{FN}\right)\left(\mathrm{TN}+\mathrm{FP}\right)\left(\mathrm{TN}+\mathrm{FN}\right)}}$$

(7) $$\text{Kappa}=\frac{P_{0}-P_{e}}{1-\mathrm{Pe}}$$

Where TP, FP, TN, and FN refer to true positive, false positive, true negative, and false negative, respectively. P_0_ denotes the relative observed agreement among raters, and P_e_ indicates the hypothesized likelihood of chance agreement.

As the graph of true positive rate (TPR) against false positive rate (FPR), receiver operating characteristic (ROC) curve reflects the diagnostic ability of a model. Area under curve (AUC) refers to the area under the ROC curve, presenting the sum measured classification performance across all possible thresholds.

### Statistical analysis

The Mann‐Whitney U test, Chi‐square test, and Fisher's exact test were employed to assess the differences in clinical characteristics between the training and the validation set. The statistical significance level was set to *P* < 0.05 for all statistical tests. R software (version 3.5.3) and SPSS 19 software (SPSS Inc., Chicago, IL) were applied to carry out the statistical analysis and figure plots. Machine learning techniques were performed with Python 3 (Python 3.6.5) based on the following package: Scikit‐learn v 0.19.1, LightGBM v2.1.0, and Optuna v0.4,23.

## Results

### Demographic features of patients

Overall, the number of patients who had advanced NSCLC met the inclusion and exclusion was 99 (average age, 52.7 ± 12.3 years), and of which sarcopenia was found in 40 patients. The participants were split into two different groups: the training cohort with 69 patients (28 with sarcopenia, 41 without sarcopenia); and the validation cohort with 30 patients (12 with sarcopenia, 18 without sarcopenia).Table 1 shows the demographic and clinical features of all patients involved in the training and validation cohorts. No significant differences were found between the training and validation sets in terms of BMI, histological subtype, TNM staging, and sarcopenia. However, there were significant differences in age and gender between the two groups.

**Table 1:** Clinical characteristics of patients with NSCLC and sarcopenia

	Training cohort	Test cohort	
	(*n* = 69)	(*n* = 30)	*P*‐value
Age (years)			
Median (range)	60.0 (40.0–86.0)	64.5 (39.0–83.0)	**<0.001** ^†^
BMI (kg/m^2^)			
Median (range)	22.55 (16.33–29.76)	22.54 (18.23–31.45)	0.756^†^
Gender (*n*/%)			
Female	30 (43.5%)	6 (20.0%)	**0.026** ^‡^
Male	39 (56.5%)	24 (80.0%)	
Sarcopenia (*n*/%)			
Sarcopenia	28 (40.6%)	12 (40.0%)	0.957^‡^
Nonsarcopenia	41 (59.4%)	18 (60.0%)	
Histological subtype (*n*/%)			
Adenocarcinoma	56 (81.2%)	23 (76.7%)	
Squamous cell carcinoma	10 (14.5%)	6 (20.0%)	0.276^§^
Mixed	3 (4.3%)		Two‐sided Pr ≤ *p* 0.276
Sarcomatoid carcinoma		1 (3.3%)	
TNM (*n*/%)			
IIIb	10 (14.5%)	4 (13.3%)	
IIIc	5 (7.2%)	2 (6.7%)	0.983^§^
IVa	16 (23.2%)	8 (26.7%)	Two‐sided Pr ≤ *p* 0.983
IVb	38 (55.1%)	16 (53.3%)	

^†^ Mann‐Whitney U test.

^‡^ Chi‐square test.

^§^ Fisher's exact test.

### Robustness analysis

**Figure 4:**
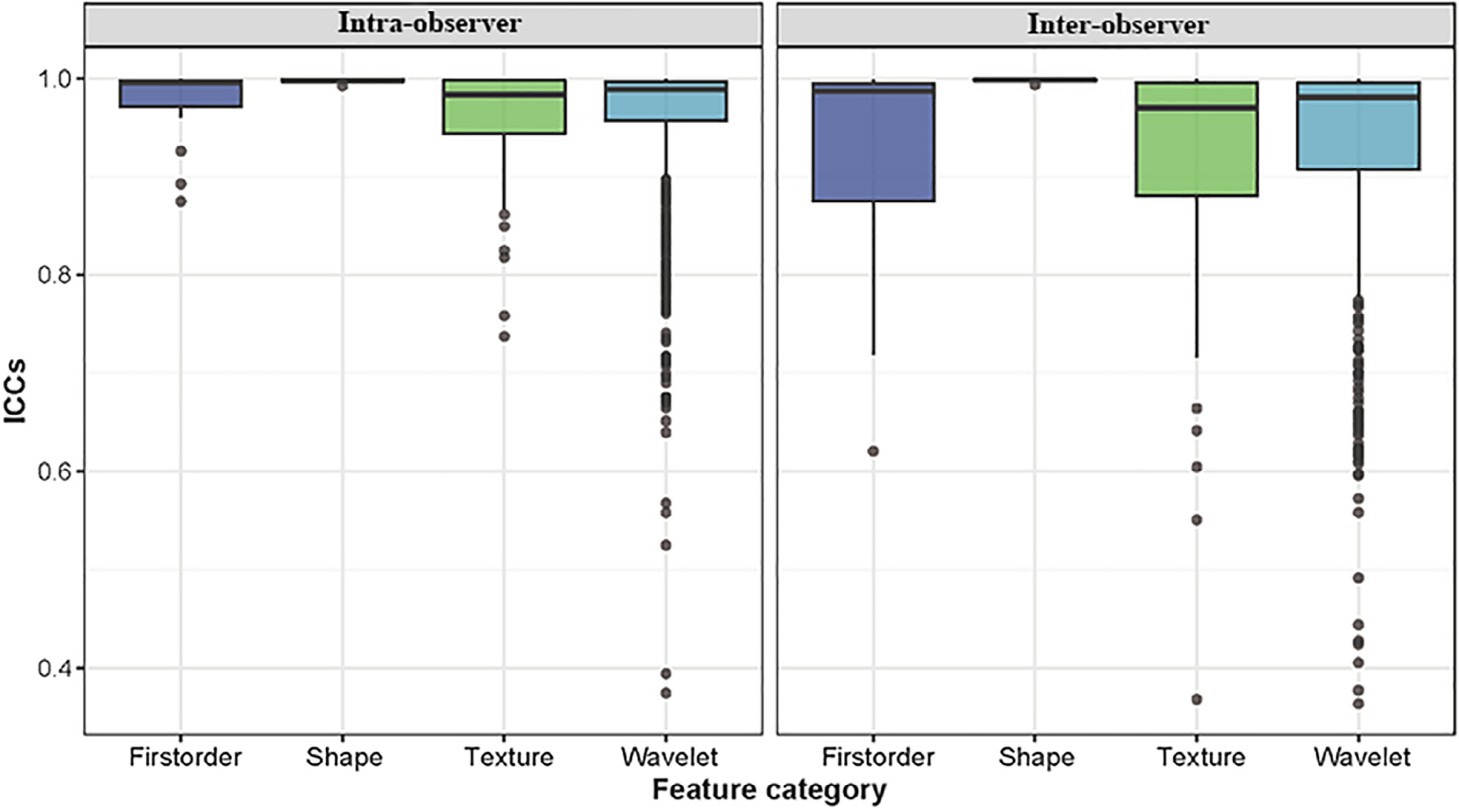
Boxplot of intra‐ and interobserver intraclass correlation coefficients (ICCs) of four radiomic feature categories.

Body composition analysis by single cross‐sectional on L3 CT imaging showed satisfactory reproducibility with good interobserver (ICC = 0.991) and intraobserver reproducibility (ICC = 0.992). The robustness of radiomic features with ICC analysis is presented in Fig 4. Among the 851 radiomic features, 783 (92.2%) which showed good inter‐ and intraobserver reproducibility (ICC ≥ 0.75) were selected, and combined with three clinic features for further ML analysis.

### Feature selection

**Figure 5:**
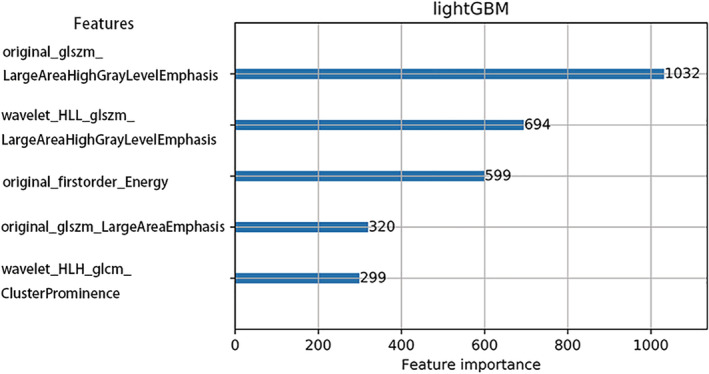
The selected key features and their importance score after feature selection.

After the feature selection procedure, five key features were selected: one First Order features, two Texture features, and two Wavelets features. The selected key features and their importance score are shown in Fig 5.

With regard to the 781 eliminated features, two features had Null value over 60%, and no feature had a single unique value. A total of 681 features were removed for having a correlation magnitude higher than 0.80. Moreover, 755 features were labeled as zero importance, and 19 features were required for the cumulative importance of 0.90.

### Model performance

**Figure 6:**
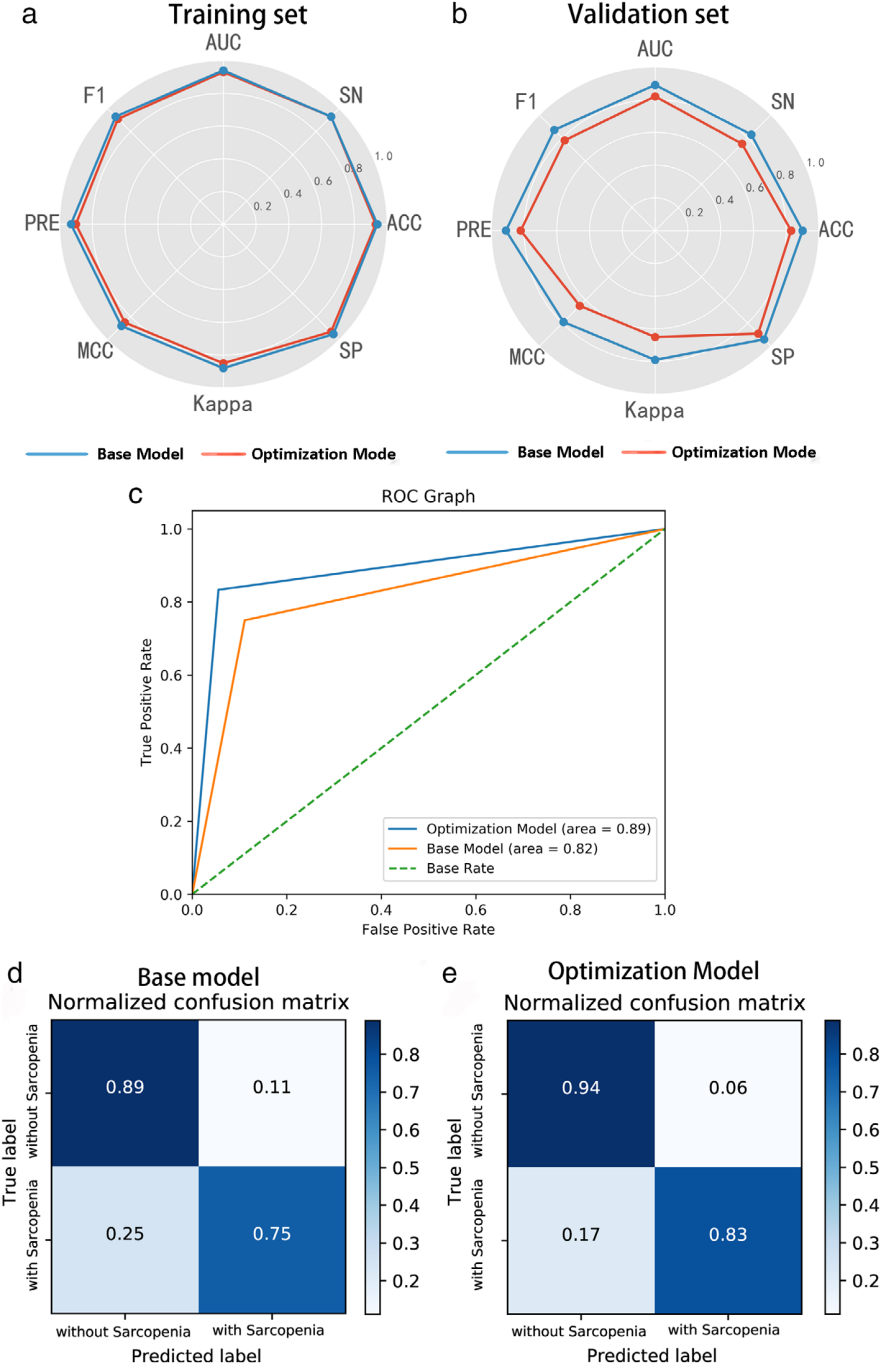
The performance of lightGBM in identifying sarcopenia. Radar plot illustrations the performance of base and optimal lightGBM model in (**a**) training and (**b**) validation set set; (**c**) ROC curves of the base and optimal lightGBM classifier in validation set; Confusion matrix with in validation set with (**d**) base; and (**e**) optimal lightGBM classifier.

According to Table 2 and Fig 6, the lightGBM model showed good performance in identifying sarcopenia in the NSCLC patients.

**Table 2:** The performance of lightGBM classifier in identifying sarcopenia

	Base model		Optimized model	
	Training set	Validation set	Training set	Validation set
Specificity	0.927	0.889	0.951	0.944
Sensitivity	0.929	0.750	0.929	0.833
Accuracy	0.928	0.833	0.942	0.900
Precision	0.897	0.818	0.929	0.909
F1‐score	0.912	0.783	0.929	0.870
AUC	0.928	0.819	0.940	0.889
MCC	0.851	0.649	0.880	0.791
Cohen's kappa	0.851	0.648	0.880	0.789

MCC, Matthew's correlation coefficient.

In the base lightGBM model with default setting, the specificity, sensitivity, accuracy, precision, f1‐score, AUC, MCC, Cohen's kappa training set and validation set were 0.927, 0.929, 0.928, 0.897, 0.912, 0.928, 0.851, 0.851; 0.889, 0.750, 0.833, 0.818, 0.783, 0.819, 0.649, 0.648, respectively. After the Bayesian hyperparameter tuning, the optimized lightGBM model achieved better prediction performance in both the training and the validation set. The corresponding values for the training and the validation set were 0.951, 0.929, 0.942, 0.929, 0.929, 0.940, 0.880, 0.880; 0.944, 0.833, 0.900, 0.909, 0.870, 0.889, 0.791, 0.789, respectively.

## Discussion

Sarcopenia was initially defined as age‐related skeletal muscle mass wasting and was reported to be caused by cancer or other underlying diseases.^2^, ^3^ There is evidence that sarcopenia is linked to a worse overall prognosis in various cancers, and the pervasiveness of sarcopenia in lung cancer is more significant compared to many other types of cancer.^18^ In previous studies, it has been shown that in NSCLC patients, the prevalence of sarcopenia varied from 43% to 46.8%.^2^, ^19^, ^20^ The pervasiveness of sarcopenia in our study was 40.4%, which is consistent with the literature.

The early diagnosis of sarcopenia can be significantly improved by the precise measurement of muscle loss associated with it. However, the lack of abdominal CT scans for lung cancer patients remains an obstacle in the diagnosis of sarcopenia.^2^ Therefore, various studies have attempted to solve this dilemma by conducting CT scans at different vertebra levels (T4, L1) for SMM estimates in NSCLC patients.^5^, ^6^, ^21^, ^22^ Although good results have been achieved, the applications were not so extensive. Herein, an accurate and reproducible classifier was constructed through the integration of a large panel of skeletal muscle CT radiomic features and high efficiency lightGBM model, to differentiate NSCLC patients with sarcopenia from those without sarcopenia, achieving high accuracy and AUC were recorded as 0.900 and 0.889 in optimal lightGBM mode.

Possessed two novel techniques: Gradient‐based One‐Side Sampling (GOSS) and Exclusive Feature Bundling (EFB), LightGBM could facilitate the training process of conventional GBDT model by over 20 times and achieve almost the identical performance in multiple experiments.^16^, ^23^ Both classification and regression tasks have been commonly performed using LightGBM.^23^, ^24^, ^25^, ^26^, ^27^ Nevertheless, the sensitivity to overfitting presents the toughest challenge to the LightGBM algorithm, particularly with regard to the small dataset.^28^ Thus, it is necessary to carefully tune the parameters of the LightGBM model.

Relative to the conventional ML algorithms that only require adjustment to two or fewer parameters, tuning lightGBM is far more complicated and makes it necessary to tune a larger number of parameters to ensure the accuracy and robustness of the model.

To optimize the 11 lightGBM parameters, the simplest method is to use the grid‐search strategy. However, a substantial amount of cost is incurred when tuning all the 11 parameters simultaneously via grid‐searching, particularly in the case of a relatively large search space. In order to address this issue, it is advisable to adopt Bayesian optimization strategies to perform hyperparameter tuning, as it has better performance on the test set and requires fewer iterations compared to grid and random searches.^29^, ^30^ Our results demonstrated that the baseline lightGBM model could be improved via Bayesian optimization by over 8.0% in ACC value, 11.1% in F‐value, 21.9% in MCC value, and 8.5% in AUC value, on the validation set, respectively, thereby verifying the effectiveness of Bayesian optimization.

Our research is not the first which has attempted to identify skeletal muscle changes with radiomics. de Jong *et al*.^9^ recently investigated the potential of skeletal muscle radiomic features to predict future muscle loss in stage IV NSCLC patients. However, the results only achieved an average AUC of 0.49 in predicting future muscle loss, and an average AUC of 0.68 in discriminatory patients who lost muscle and those who maintained muscle mass. There was a better performance in our study which used skeletal muscle radiomics to identify sarcopenia in patients with advanced NSCLC. The better result would attribute to a single institute experiment, pretreatment CT scan, and the powerful lightGBM model in our research.

This study had several limitations. First, we retrospectively assessed sarcopenia by muscle mass depletion analysis based on calculated L3‐SMI, which is a routinely and widely used method,^13^, ^20^ and the absence of impaired muscle function could hinder the correct identification of sarcopenia;^31^, ^32^ however, additional prospective research will assist in solving this problem. Second, all the datasets herein were acquired from the same clinical center with the same PET/CT scanner. In the future, it will be necessary to conduct a multicenter study with a greater sample size.

In conclusion, CT based radiomics has the potential to identify sarcopenia in NSCLC patients with lightGBM classifier. The optimal lightGBM model via Bayesian hyperparameter tuning demonstrated better performance.

## Disclosure

The authors declare that they have no conflicts of interest.

## Supporting information


**Table S1.** Summary of 851 radiomics features
**Table S2.** Hyperparameter settings of lightGBM used for the Bayesian optimization
