# Supplementary material for: Identifying sarcopenia in advanced non‐small cell lung cancer patients using skeletal muscle CT radiomics and machine learning
Source: Thorac Cancer. 2020 Aug 6;11(9):2650–9. doi: 10.1111/1759-7714.13598 (PMC7471037; doi:10.1111/1759-7714.13598)
Supplement: Supplementary file 1 — Table S1. Summary of 851 radiomics features Table S2. Hyperparameter settings of lightGBM used for the Bayesian optimization [file TCA-11-2650-s001.docx]

**Supplementary Materials**

| **Table S1. Summary of 851 radiomics features** | | | | |  |  |
| --- | --- | --- | --- | --- | --- | --- |
| Feature category | | | |  |  | Feature numbers |
| **Shape Features** | | |  |  |  | ***14*** |
|  | *1)* | *Elongation* | *2)* | *Flatness* | *3)* | *Least Axis Length* |
|  | *4)* | *Maximum 2D Diameter Row* | *5)* | *Voxel Volume* | *6)* | *Major Axis Length* |
|  | *7)* | *Maximum 2D Diameter Slice* | *8)* | *Maximum 3D Diameter* | *9)* | *Mesh Volume* |
|  | *10)* | *Minor Axis Length* | *11)* | *Sphericity* | *12)* | *Surface Area* |
|  | *13)* | *Surface Volume Ratio* | *14)* | *Maximum 2D Diameter Column* |  |  |
| **First Order Features** | | | |  |  | ***18*** |
|  | *1)* | *10th Percentile* | *2)* | *90th Percentile* | *3)* | *Energy* |
|  | *4)* | *Entropy* | *5)* | *Interquartile Range* | *6)* | *Kurtosis* |
|  | *7)* | *Maximum* | *8)* | *Minimum* | *9)* | *Mean* |
|  | *10)* | *Median* | *11)* | *Uniformity* | *12)* | *Range* |
|  | *13)* | *Total Energy* | *14)* | *Variance* | *15)* | *Skewness* |
|  | *16)* | *Root Mean Squared (RMS)* | *17)* | *Mean Absolute Deviation(MAD)* |  |  |
|  | *18)* | *Robust Mean Absolute Deviation (rMAD)* |  |  |  |  |
| **Texture** | | | |  |  | ***75*** |
|  | ***Gray Level Co-occurrence Matrix (GLCM) Features*** | | | |  | *24* |
|  | *1)* | *Autocorrelation* | *2)* | *Joint Average* | *3)* | *Cluster Prominence* |
|  | *4)* | *Cluster Shade* | *5)* | *Cluster Tendency* | *6)* | *Contrast* |
|  | *7)* | *Correlation* | *8)* | *Difference Average* | *9)* | *Difference Entropy* |
|  | *10)* | *Difference Variance* | *11)* | *Joint Energy* | *12)* | *Joint Entropy* |
|  | *13)* | *Maximum Probability* | *14)* | *Sum of Squares* | *15)* | *Sum Entropy* |
|  | *16)* | *Inverse Variance* | *17)* | *Inverse Difference (ID)* | *18)* | *Sum Average* |
|  | *19)* | *Inverse Difference Moment (IDM)* | *20)* | *Inverse Difference Moment Normalized (IDMN)* | | |
|  | *22)* | *Inverse Difference Normalized (IDN)* | *21)* | *Informal Measure of Correlation (IMC) 1* | | |
|  | *23)* | *Maximal Correlation Coefficient (MCC)* | *24)* | *Informal Measure of Correlation (IMC) 2* | | |
|  | ***Gray Level Dependence Matrix (GLDM) Features*** | | | |  | *14* |
|  | *1)* | *Dependence Entropy (DE)* | *2)* | *Dependence Non-Uniformity (DN)* | | |
|  | *4)* | *Dependence Variance (DV)* | *3)* | *Dependence Non-Uniformity Normalized (DNN)* | | |
|  | *5)* | *Gray Level Non-Uniformity (GLN)* | *6)* | *Large Dependence Low Gray Level Emphasis (LDLGLE)* | | |
|  | *7)* | *Gray Level Variance (GLV)* | *8)* | *Large Dependence High Gray Level Emphasis (LDHGLE)* | | |
|  | *9)* | *High Gray Level Emphasis (HGLE)* | *10)* | *Large Dependence Low Gray Level Emphasis (LDLGLE)* | | |
|  | *11)* | *Large Dependence Emphasis (LDE)* | *12)* | *Large Dependence High Gray Level Emphasis (LDHGLE)* | | |
|  | *13)* | *Low Gray Level Emphasis (LGLE)* | *14)* | *Small Dependence Emphasis (SDE)* | | |
|  | ***Gray Level Run Length Matrix (GLRLM) Features*** | | | | | *16* |
|  | *1)* | *Short Run Emphasis (SRE)* | *2)* | *Short Run Low Gray Level Emphasis (SRLGLE)* | | |
|  | *4)* | *Long Run Emphasis (LRE)* | *3)* | *Short Run High Gray Level Emphasis (SRHGLE)* | | |
|  | *5)* | *Gray Level Non-Uniformity (GLN)* | *6)* | *Long Run Low Gray Level Emphasis (LRLGLE)* | | |
|  | *7)* | *Run Percentage (RP)* | *8)* | *Long Run High Gray Level Emphasis (LRHGLE)* | | |
|  | *9)* | *Run Length Non-Uniformity (RLN)* | *10)* | *Gray Level Non-Uniformity Normalized (GLNN)* | | |
|  | *11)* | *Gray Level Variance (GLV)* | *12)* | *Run Length Non-Uniformity Normalized (RLNN)* | | |
|  | *13)* | *Run Variance (RV)* | *14)* | *Low Gray Level Run Emphasis (LGLRE)* | | |
|  | *15)* | *Run Entropy (RE)* | *16)* | *High Gray Level Run Emphasis (HGLRE)* | | |
|  | ***Gray Level Size Zone Matrix (GLSZM) Features*** | | | |  | *16* |
|  | *1)* | *Small Area Emphasis (SAE)* | *2)* | *Gray Level Non-Uniformity Normalized (GLNN)* | | |
|  | *4)* | *Large Area Emphasis (LAE)* | *3)* | *Size-Zone Non-Uniformity Normalized (SZNN)* | | |
|  | *5)* | *Gray Level Non-Uniformity (GLN)* | *6)* | *Small Area Low Gray Level Emphasis (SALGLE)* | | |
|  | *7)* | *Size-Zone Non-Uniformity (SZN)* | *8)* | *Small Area High Gray Level Emphasis (SAHGLE)* | | |
|  | *9)* | *Zone Percentage (ZP)* | *10)* | *Large Area Low Gray Level Emphasis (LALGLE)* | | |
|  | *11)* | *Gray Level Variance (GLV)* | *12)* | *Large Area High Gray Level Emphasis (LAHGLE)* | | |
|  | *13)* | *Zone Variance (ZV)* | *14)* | *Low Gray Level Zone Emphasis (LGLZE)* | | |
|  | *15)* | *Zone Entropy (ZE)* | *16)* | *High Gray Level Zone Emphasis (HGLZE)* | | |
|  | ***Neighbouring Gray Tone Difference Matrix (NGTDM) Features*** | | | |  | *5* |
|  | *1)* | *Coarseness* | *2)* | *Contrast* | *3)* | *Busyness* |
|  | *4)* | *Complexity* | *5)* | *Strength* |  |  |
| **Wavelet Group** | | | | |  | ***744*** |
|  | *LLL HLL LHL HHL LLH HLH LHH HHH decomposition for intensity and texture features* | | | | | |
|  |  |  |  | Total feature number | | ***851*** |

| **Table S2.** Hyperparameter settings of lightGBM used for the Bayesian optimization. | | |
| --- | --- | --- |
| **Parameters** | **Search space** | **Optimal value** |
| learning_rate | (1e-6,1e-1,'log-uniform') | 0.205166917 |
| lambda_l1 | (1e-6,1e1,'log-uniform') | 2.33E-05 |
| lambda_l2 | (1e-6,1e1,'log-uniform') | 0.537458844 |
| bagging_fraction | (0.4, 1.0,'uniform') | 0.840245556 |
| feature_fraction | (0.4, 1.0,'uniform') | 0.429391965 |
| bagging_freq | (1, 10,'int') | 9 |
| min_child_samples | (1,100,'int') | 21 |
| max_depth | (1, 20,'int') | 17 |
| num_leaves | (2,256,'int') | 49 |
| max_bin | (1,500,'int') | 167 |
| n_estimators | (100, 1000,'int') | 303 |
